# Cognitive fatigue in relation to depressive symptoms after treatment for childhood cancer

**DOI:** 10.1186/s40359-020-00398-1

**Published:** 2020-04-10

**Authors:** Elin Irestorm, Ingrid Tonning Olsson, Birgitta Johansson, Ingrid Øra

**Affiliations:** 1 Children’s Hospital, Neuropaediatrics, Skåne University Hospital, SE-221 85 Lund, Sweden; 2 Faculty of Medicine, Department of Clinical Sciences Lund, Paediatrics, Lund University, Lund, Sweden; 3 Faculty of Social Sciences, Department of Psychology, Lund University, Lund, Sweden; 4 Institute of Neuroscience and Physiology, University of Gothenburg, Gothenburg, Sweden; 5 Children’s Hospital, Paediatric Oncology, Skåne University Hospital, Lund, Sweden

**Keywords:** Cognitive fatigue, Brain tumour, Acute lymphoblastic leukaemia, Depression

## Abstract

**Background:**

Cognitive fatigue after childhood cancer is frequently overlooked despite guidelines recommending follow-up, and might be mistaken for depression due to overlapping symptoms. Our objectives were: 1) to examine ratings of fatigue in survivors of paediatric brain tumours (BT) and acute lymphoblastic leukaemia (ALL) compared to healthy controls, 2) to examine the relationship between symptoms of depression and cognitive fatigue, and 3) to evaluate parent-child concordance in ratings of cognitive fatigue.

**Methods:**

Survivors of BT (*n* = 30), survivors of ALL (n = 30), and healthy controls (*n* = 60) aged 8–18 years completed the Pediatric Quality of Life Multidimensional Fatigue Scale and the Beck Youth Inventories. Associations between cognitive fatigue, diagnosis and depression were assessed with general linear modelling. Group differences were analysed using the Kruskal–Wallis test. Parent-child concordance was investigated with internal consistency reliability.

**Results:**

Cognitive fatigue was prevalent in 70% of survivors of BT survivors and in 30% of survivors of ALL. Diagnosis was the main predictor of cognitive fatigue (*p* < .001, η_p_^2^ = 0.178), followed by depression (*p* = .010, η_p_^2^ = 0.080). Survivors of BT reported significantly more fatigue than healthy controls on all fatigue subscales. While they also expressed more symptoms of depression, we found no evidence for an interaction effect. Parent-child concordance was moderate to good among survivors, but poor for controls.

**Conclusions:**

Survivors of BT and ALL suffer from cognitive fatigue, with survivors of BT expressing more problems. Cognitive fatigue and depression should be assessed in survivors of childhood cancer using both self-rated and proxy-rated measures, and appropriate interventions offered.

## Background

Cognitive fatigue (sometimes referred to as mental fatigue or brain fatigue) is frequently overlooked as a long-term sequela to paediatric cancer diagnosis and treatment [1, 2]. In previous studies, cancer-related fatigue in general was consistently found to be one of the most prevalent and distressing symptoms in childhood cancer survivors [2–4]. Furthermore, there is a need to discriminate between physical and cognitive fatigue amongst these young survivors [5]. This is important as overlapping symptoms might cause cognitive fatigue to be mistaken for depression, potentially leading to inadequate treatment. Cognitive fatigue includes a spectrum of deficits affecting motivation, emotion, cognition and behaviour [6]. The sequela is often associated with difficulties in maintaining attention and information processing, as well as memory and executive functions [6–10]. Mental exhaustion caused by sensory stimulation and/or prolonged cognitive tasks is a characteristic symptom [11]. A specific diurnal pattern is also a clinical feature of this sequela, where performance decreases, and sensitivity to sound (and in some patient groups also sensitivity to light), continuously increases during the day [12–14].

Cognitive fatigue in adults is associated with head trauma, stroke, sepsis, multiple sclerosis, Parkinson’s disease, meningitis, encephalitis, brain tumours and breast cancer [11, 15–19]. Considering the current knowledge regarding cognitive fatigue related to adults with cancer or acquired brain injuries, as well as several other neurological conditions and disorders, this possible long-term complication should also be assessed and monitored in younger patients. In addition, the overlap between cognitive fatigue and depressive symptoms, such as mood swings and irritability, and problems associated with sleep, memory and attention [9, 13] indicates that cognitive fatigue after cancer may be mistaken for depression if not thoroughly assessed. Previous studies have demonstrated that depression and cognitive fatigue are separate constructs [13, 20], and this distinction is important in ensuring that the correct therapeutic strategies are instigated.

One of the main reasons for differentiating between cognitive and physical fatigue is the theory of diverse origins of the conditions [21–23]. Cognitive fatigue is suggested to be associated with neuroinflammation [24], and believed to be the result of down-regulation of glutamate transmission and dysfunction of the astroglial cells in removing glutamate from the extracellular space. This could cause impaired neuronal function and lead to exhaustion after high mental load [24, 25]. If these processes are not fully restored, this could cause unspecific neuronal signalling and lack of energy, resulting in further prolongation of the time to restoration [11].

Although cognitive fatigue is associated with disorders of the central nervous system [26], it is also frequently observed after breast cancer treatment [27, 28], indicating that chemotherapy may initiate or cause this dysfunction. It has been concluded in systematic reviews and meta-analyses of cognitive deficits after treatment for paediatric acute lymphoblastic leukaemia (ALL), that although children treated with radiotherapy suffer the worst deficits, children treated with chemotherapy alone were also affected [29, 30]. Most chemotherapeutic molecules do not pass the blood-brain barrier, nevertheless, they may still cause toxicity in the brain indirectly through proinflammatory cytokine pathways. Proinflammatory cytokines impair astroglial glutamate uptake, and increased levels of proinflammatory cytokines have been reported in disorders associated with cognitive fatigue [24, 31], as well as after chemotherapy for paediatric ALL [30].

The Pediatric Quality of Life (PedsQL™) module Multidimensional Fatigue Scale is an instrument for measuring cognitive fatigue in children and teenagers [32]. Specific versions are available divided by age groups from 2 to 25 years [33, 34], and the instrument has been applied to assess the effects of several conditions and diseases in children, such as cancer [33], sickle cell disease [35] and rheumatology [36]. In a recent systematic review of existing fatigue instruments, the authors concluded that the PedsQL™ Multidimensional Fatigue Scale was the only instrument among those studied with strong evidence of reliability [37]. Reliability and validity have been found to be stable across languages, age and gender [32, 38, 39].

Cognitive fatigue in children with brain tumours (BTs) has been compared to that in children with ALL using the PedsQL™ Multidimensional Fatigue Scale, showing that the latter group was less affected by cognitive fatigue [40]. However, the overlap with depression was not investigated, the study lacked a control group, and only the parent-proxy version of PedsQL™ was used. Considering the limited knowledge regarding cognitive fatigue after treatment for childhood cancer, further research on paediatric cancer survivors is warranted. The objectives of the current study were: 1) to examine ratings of fatigue in survivors of BT and ALL compared to healthy controls, 2) to examine the relationship between symptoms of depression and cognitive fatigue, and 3) to evaluate parent-child concordance in ratings of cognitive fatigue.

## Methods

### Participants

Children diagnosed with BT or ALL at Skåne University Hospital, Lund, Sweden, aged between 8 and 18 years, and who completed treatment more than 2 years ago were eligible to participate. Exclusion criteria were: 1) non-proficiency in Swedish, 2) diagnosis of intellectual disability, or 3) diagnosis of Down’s syndrome. A total of 65 survivors were eligible. Three families declined to participate, and a further two were excluded due to relapse after inclusion, leaving 60 survivors in the study. A control group of 60 healthy children, 8–18 years of age, was recruited from the general community.

### Questionnaires

In this study, both parent-proxy and self-reported versions of the PedsQL™ were administered. The Multidimensional Fatigue Scale is divided into general fatigue, sleep/rest fatigue and cognitive fatigue, from which a composite total fatigue scale is obtained. The questions cover the frequency of different symptoms of fatigue and are scored on a 5-point Likert scale from 0 to 4 (i.e., 0 = “never”, 1 = “almost never”, 2 = “sometimes”, 3 = “often”, 4 = “almost always”). The answers are reversed and linearly transformed into a scale from 0 to 100, where higher scores indicate less problems. No cut-off is provided in PedsQL™, and a score below the 10th percentile of the control group was therefore used as an indicator of cognitive fatigue, as established for the self-rating questionnaires used in the Childhood Cancer Survivor Study protocols [41].

The Beck Youth Inventories Depression subscale [42] was used for the assessment of depressive symptoms. The questions cover the frequency of different symptoms of depression, and are scored on a 4-point Likert scale from 0 to 3 (i.e., 0 = “never”, 1 = “sometimes”, 2 = “often”, 3 = “always”). Raw scores from this self-reported questionnaire were transformed into percentiles, and higher scores on this scale indicate more symptoms of depression. According to Swedish norms, scores up to the 74th percentile are regarded as average, scores between the 75th and 89th percentile are regarded as elevated, and scores above the 90th percentile are regarded as highly elevated [42]. Results within the elevated or highly elevated range do not constitute a diagnosis of depression but should be interpreted as a measure of self-perceived symptoms.

### Procedures

For survivors, data were collected at the scheduled follow-up visits at the university hospital 2–6 years after the end of treatment. Parents and children completed the questionnaires independently of each other and did not see each other’s answers. A member of the research team was available to assist the child if needed. Controls were recruited from the general community through advertisement, but otherwise the procedure was identical. Families were not offered financial compensation for participation, but the child received a symbolic gift. Data on diagnosis and treatment were retrieved from medical records.

### Statistical methods

SPSS version 25 was used for statistical analysis. As several of the parent-proxy scales violated assumptions of parametric tests, group differences for the results obtained with PedsQL™ were analysed with the non-parametric Kruskal–Wallis test. The Mann–Whitney U test was used for post hoc analysis of the significant differences, and effect sizes were calculated for the pairwise tests. Parent-child rating concordance was investigated by examining internal consistency reliability using Cronbach’s alpha and the intraclass correlation coefficient between the self-rating and parent-proxy versions. For Cronbach’s alpha, scores between 0.70 and 0.90 were considered satisfactory [43]. For intraclass correlation coefficient, values less than 0.50 were considered to indicate poor reliability, between 0.50 and 0.75 to indicate moderate reliability, and between 0.75 and 0.90 to indicate good reliability [44]. Gender differences were investigated with the Mann–Whitney U test. A general linear model was used to analyse the effect of diagnosis and symptoms of depression on the PedsQL™ self-rating cognitive fatigue subscale. A factorial ANOVA was chosen in order to examine association between the two variables diagnosis and depression. Diagnosis included three levels (controls, survivors of ALL, survivors of BT), and depression included three levels (average symptoms, elevated symptoms, highly elevated symptoms).

## Results

### Participant demographics and clinical characteristics

Average age and standard deviations (SD) at assessment was similar across all three groups: 13.31 (*SD* = 2.80) years for survivors of BT, 12.47 (*SD* = 2.96) years for survivors of ALL and 12.18 (*SD =* 2.84) years for the controls (Table 1). The age range in all three groups was 8–18 years at the time of assessment. Average time from last treatment was 3.28 (*SD* = 1.94) years for survivors of BT and 3.90 (*SD* = 1.86) years for survivors of ALL. Average time since diagnosis was 4.67 (*SD* = 2.66) years for survivors of BT and 7.01 (*SD* = 2.04) years for survivors of ALL, due to the longer treatment protocols for ALL. Five survivors of BT and 4 survivors of ALL had previously been treated for a relapse. Seventy-three percent of the ALL survivors were male, while 50% of the survivors of BT and 50% of the controls were male.

**Table 1:** Participant characteristics

	BT	ALL	Controls
Total number	30	30	60
Mean age at inclusion, years (SD)	13.31 (2.80)	12.47 (2.96)	12.18 (2.84)
Median age at inclusion, years	12.67	12.16	12.00
Age range, years	8.25–18.11	8.19–18.02	8.00–17.95
Mean time since first diagnosis (SD)	4.67 (2.66)	7.01 (2.04)	
Mean time since end of last treatment (SD)	3.28 (1.94)	3.90 (1.86)	
Number treated for relapse, n (%)	5 (16.7)	4 (13.3)	
Gender, n (%)			
Female	15 (50)	8 (26.7)	31 (51.7)
Male	15 (50)	22 (73.3)	29 (48.3)

Abbreviations: *BT* brain tumour survivors; *ALL* acute lymphoblastic leukaemia survivors

### Measures of fatigue

**Fig. 1:**
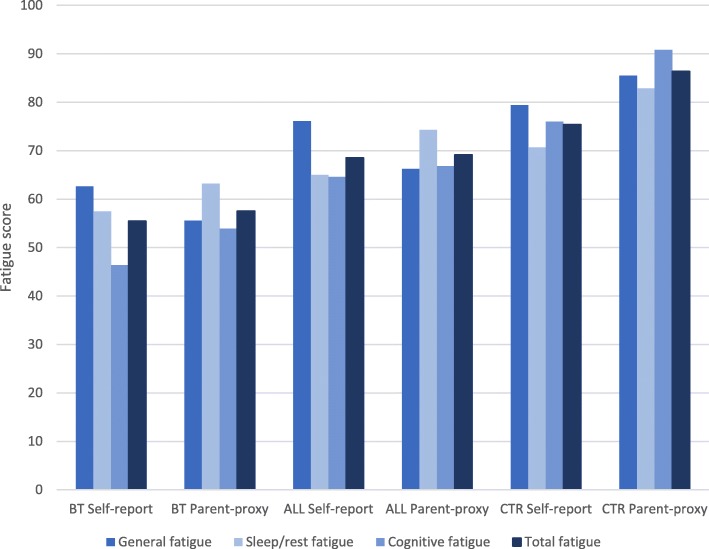
Results for the four different subscales from PedsQL™ Multidimensional Fatigue Scale. Maximum score is 100, where lower scores indicate more problems. Abbreviations: BT, brain tumour survivors; ALL, acute lymphoblastic leukaemia survivors, CTR; controls

Mean fatigue scores for the different groups and subscales are visualised in Fig. 1. Controls scored highest of all three groups (indicating less problems) for all subscales. Means scores ranged from 70.69 (*SD* = 15.67) to 90.83 (*SD* = 13.3) for controls, with higher scores for the parent-proxy reports than the self-reports. The mean values were very close to those previously reported in healthy controls in a European sample [38].

Survivors of BT scored lowest in all measures of fatigue, both self-reported and parent-proxy reported. Mean scores ranged from 46.39 (*SD* = 29.29) to 63.19 (*SD* = 19.17) for survivors of BT, and the results differed significantly from controls in all eight fatigue measures. Parents scored less problems than survivors for all measures except general fatigue.

Survivors of ALL consistently scored higher than survivors of BT, but lower than controls, with mean values ranging from 64.58 (*SD* = 25.11) to 76.11 (*SD* = 13.29). The pattern was identical to that of survivors of BT, as parents scored less problems than survivors for all measures except general fatigue.

Effect sizes were largest when survivors of BT were compared to the controls (Table 2). However, as visualised in Fig. 1, comparing effect sizes for the total fatigue scale might not be meaningful as the pattern differed between the different groups. For the two groups of survivors, parents rated less problems with sleep fatigue than the children did, but since the survivors rated more problems with general fatigue, this affected the composite total fatigue scale (which is the mean value of the 3 subscales). While children rated greater problems than parents, the differences were smaller for the cancer survivors than for the controls. Seventy percent of survivors of BT had scores below the poorest 10% of the control group for cognitive fatigue (corresponding to a score below 55 on a scale 0–100), whereas 30% of survivors of ALL scored below this cut-off. No significant gender differences were found for any of the PedsQL™ subscales.

**Table 2:** Child self-reported and parent proxy-reported fatigue in survivors and controls, together with the results of Mann–Whitney post hoc test with effect sizes

	Brain tumours			Acute lymphoblastic leukaemia			Controls		post hoc analysis	*r*	
**Child self-reported**	*M*	*SD*	95% CI	*M*	*SD*	95% CI	*M*	*SD*	95% CI		
General fatigue	62.63	24.76	53.38–71.85	76.11	13.29	70.13–82.09	79.44	13.63	75.92–82.97	BT vs. CTR	−.33
Sleep/rest fatigue	57.50	22.44	49.12–65.88	64.99	17.63	58.42–71.58	70.69	15.67	66.65–74.74	BT vs. CTR	−.29
Cognitive fatigue	46.39	29.29	35.45–57.33	64.58	25.11	55.20–73.96	76.04	14.75	72.23–79.85	BT vs. CTR BT vs. ALL	−.50 −.32
Total fatigue	55.50	20.93	47.69–63.32	68.56	16.47	62.41–74.72	75.39	12.62	72.13–78.65	BT vs. CTR	−.45
**Parent-proxy-reported**	*M*	*SD*	95% CI	*M*	*SD*	95% CI	*M*	*SD*	95% CI		
General fatigue	55.56	21.17	47.65–63.46	66.25	23.27	57.56–74.94	85.49	14.03	81.86–89.11	BT vs. CTR ALL vs. CTR	−.62 −.39
Sleep/rest fatigue	63.19	19.17	56.03–70.35	74.30	19.98	66.80–81.81	82.86	14.55	79.10–86.62	BT vs. CTR BT vs. ALL	−.51 −.32
Cognitive fatigue	53.89	25.45	44.39–63.39	66.81	25.01	57.33–76.29	90.83	13.37	87.38–94.29	BT vs. CTR ALL vs. CTR	−.67 −.50
Total fatigue	57.55	18.87	50.50–64.59	69.12	20.56	61.39–76.85	86.39	11.06	83.34–89.44	BT vs. CTR ALL vs. CTR	−.67 −.42

### Self-rated symptoms of depression and cognitive fatigue

Forty percent of BT survivors, 30% of ALL survivors, and 23% of controls reported elevated or highly elevated levels of depressive symptoms (Table 3). A factorial ANOVA was conducted to compare the effects of diagnosis and depression, and the interaction effect diagnosis*depression, on PedsQL™ self-rate cognitive fatigue subscale as the dependent variable. Effect sizes were interpreted as medium if above 0.06 and large if above 0.14 [45]. Significant results were found for both diagnosis (F_2,111_ = 11.98, *p* < .001 η_p_^2^ = 0.178) and symptoms of depression (F_2,111_ = 4.82, *p* = .010, η_p_^2^ = 0.080). A medium effect size was found for depression and a large for diagnosis. The interaction effect (F_4,111_ = 0.84, *p* = .505, η_p_^2^ = 0.029) was not significant. Hence, survivors of BT did not report more cognitive fatigue because of more depressive symptoms. The variance explained by the model was 0.312 (adjusted R^2^ = 0.262). Tukey was used for post hoc analysis. Survivors of BT scored significantly lower than both survivors of ALL and control. Mean difference between survivors of BT and ALL was − 18.19, 95% CI [− 31.30; − 5.01]. Mean difference between survivors of BT and controls was − 29.65, 95% CI [− 41.01; − 18.30]. Study participants with highly elevated symptoms of depression scored significantly more cognitive fatigue than participants with average symptoms of depression, with a mean difference of − 17.51, 95% CI [− 29.89; − 5.14].

**Table 3:** Beck Youth Inventories: self-reported depressive symptoms

	BT	ALL	Controls
Total, n (%)	30 (100)	30 (100)	60 (100)
Symptom levels, n (%)			
Average symptoms	18 (60.0)	21 (70.0)	46 (76.7)
Elevated symptoms	4 (13.3)	3 (10.0)	7 (11.7)
Highly elevated symptoms	8 (26.7)	6 (20.0)	7 (11.7)

Abbreviations: *BT* brain tumour survivors; *ALL* acute lymphoblastic leukaemia survivors

### Internal consistency reliability

Children reported more problems than their parents on most measures, resulting in lower mean scores for the self-reported values than the parent-proxy values. Parent-child concordance was better for survivors than for controls. Similarly, intraclass correlations were poor for the controls, but moderate to good for the survivors. All subscales (except sleep fatigue in the self-rated version) showed satisfactory reliability, with Cronbach’s alpha exceeding 0.70 for the total sample of survivors and controls (Table 4).

**Table 4:** Internal consistency reliability for the PedsQL™ Multidimensional Fatigue Scale

	BT	ALL	CTR	Total
**Child self-reported**	α	α	α	α
General fatigue	0.853	0.483	0.721	0.786
Sleep/rest fatigue	0.792	0.580	0.573	0.666
Cognitive fatigue	0.926	0.919	0.748	0.910
Total fatigue	0.911	0.851	0.850	0.896
**Parent-proxy-reported**	α	α	α	α
General fatigue	0.799	0.891	0.846	0.892
Sleep/rest fatigue	0.701	0.805	0.683	0.792
Cognitive fatigue	0.933	0.928	0.877	0.951
Total fatigue	0.920	0.938	0.859	0.948
**Parent-child concordance**	ICC	ICC	ICC	ICC
General fatigue	0.489	0.493	0.416	0.564
Sleep/rest fatigue	0.591	0.614	0.282	0.522
Cognitive fatigue	0.776	0.589	0.162	0.656
Total fatigue	0.740	0.706	0.313	0.679

Abbreviations: *BT* brain tumour survivors; *ALL* acute lymphoblastic leukaemia survivors; *CTR* Controls; *α* Cronbach’s coefficient alpha; *ICC* Intraclass Correlation Coefficient

## Discussion

This is the first study to examine cognitive fatigue in a Swedish sample of childhood cancer survivors, and to compare survivors of BT and ALL with healthy controls. It is also the first study to investigate the relationship between symptoms of depression and cognitive fatigue. We found that cognitive fatigue was common amongst survivors, but survivors of BT expressed more problems than survivors of ALL. While diagnosis was the strongest predictor, we also found symptoms of depression to be associated with cognitive fatigue. The outcomes of our study support the recommendation that multidimensional fatigue scales should be used in follow-up care for survivors of childhood cancer under 18 years of age. While both survivors of ALL and BT reported more symptoms of depression than healthy controls, we observed no evidence of an interaction effect. The results presented here are thus in accordance with those from previous studies, demonstrating that cognitive fatigue and depression are different constructs despite having overlapping symptoms [13, 20]. Previous studies reported a higher incidence of males in both paediatric ALL and BT [46–48], but the sex distribution in the current study was more skewed towards males than expected. However, in line with previous research [32] we found no gender differences regarding response patterns.

Diagnosis was the strongest predictor for all measures on the PedsQL™ Multidimensional Fatigue Scale. Controls consistently scored highest on all scales (indicating less problems), and the results for the controls were very similar to those reported in a Dutch sample of healthy controls [38]. In line with previous research, survivors of BT reported significantly more cognitive fatigue than ALL survivors. However, some ALL survivors also experienced cognitive fatigue. These individuals should be identified in the clinical setting, and future research should aim to elucidate medical predictors for this patient group. Self-rated symptoms of depression were associated with cognitive fatigue in the current study, and 40% of survivors of BT and 30% of survivors of ALL reported elevated or highly elevated symptoms of depression. Thus, it is important to consider mental health status during follow-up of these patient groups. We found no evidence for an interaction effect between cancer diagnosis and symptoms of depression. This implies that the higher levels of cognitive fatigue reported by BT survivors cannot be explained by more symptoms of depression in this group. Depression and cognitive fatigue therefore seem to be clinically different conditions, as concluded in previous studies [13, 20]. However, as there is an overlap between symptoms, it is important to differentiate between them, in both research and the clinical setting.

For survivors of ALL, comparisons with the control group revealed different results for the self-rate and parent-proxy version. For the self-rate version, cognitive fatigue was the only subscale were the survivors differed significantly from controls. For the parent proxy-report version, significant differences were found for two subscales and the composite total scale. Concordance between self- and proxy-rated measures has been poor in previous studies, and it has been recommended that the two scores should not be compared directly [37]. We observed that parents consistently rated less problems than children, which is cause for concern. This might indicate that parents under-report problems, but it is also possible that children over-report them. A study on children and adolescents coping with cancer reported that the percentage of patients with elevated symptoms of depression/anxiety was twice as high in the self-reports than in the parent-proxy reports [49]. Previous research on the subject of parent-child agreement have also found that agreement is higher for externalising than internalising problems [50], and that parent-child relationship is a stronger predictor for agreement than gender of the child or sociodemographic factors [51]. In similarity with depression, fatigue is an internalising rather than externalising problem. It is therefore more plausible that the low agreement was caused by parents under-reporting rather than children over-reporting symptoms.

Parent-child concordance was moderate to good amongst survivors, while we observed a low interrater reliability between parent and child reports for the healthy controls. Poor parent-child concordance for controls has also been reported in previous studies utilizing the PedsQL™ Multidimensional Fatigue Scale [38, 39]. Survivors of BT having the highest concordance of all three groups, followed by survivors of ALL, could be caused by the severity of the disabilities associated with these two diagnoses. As disabilities entail a different type of parental involvement this could explain why survivors had a higher parent-child concordance. This is in line with research reporting parent-child relationship as a strong predictor for agreement [51], and does not contradict the finding regarding parents under-reporting problems. Parents under-reporting children’s fatigue is a phenomenon that has been demonstrated in several studies applying the PedsQL™ Multidimensional Fatigue Scale to other patient groups [35, 38, 52]. These observations provide further evidence that these reports cannot be used interchangeably, and that caution should be exercised when comparing the results of studies using different measures. This is important in both research and in clinical assessments of individual patients, as children and parents may differ in their perceptions of possible deficits. The two forms can also be seen as different types of information sources, with both adding valuable information from separate perspectives.

While this study is the first to investigate the relationship between cognitive fatigue and symptoms of depression in survivors of childhood cancer, utilising instruments with high reliability and validity, the relatively small sample size is a limitation. While almost all eligible survivors agreed to participate, the study cohort was too small to allow comparisons between treatment modalities. Future studies should investigate the effects of surgery, radiotherapy and chemotherapeutic agents, alone or in combination, to further clarify the contribution of treatment modality to cognitive fatigue. Another important research area that remains to be investigated is the relationship between cognitive fatigue and cognitive function.

## Conclusions

Based on the results presented here, we conclude that cognitive fatigue is prevalent in survivors of both BT and ALL, even after adjusting for symptoms of depression. Not only survivors of BT, but also survivors of ALL experience cognitive fatigue, although to a lesser extent. While effect sizes were large for diagnosis as a predictor for cognitive fatigue, the differences between the two groups might not be clinically relevant as there are individual survivors of ALL suffering from this long-term deficit. Although more research is needed regarding medical predictors (for example type of treatment) on a group level to identify risk factors, the impact on these survivors and their families must be considered. Hence, these children and adolescents must be properly identified and assessed. Both cognitive fatigue and mental health status should be included and evaluated in the follow-up programmes of childhood cancer survivors, and appropriate interventions offered when needed. The use of multidimensional fatigue scales facilitates detection of cognitive fatigue and should be considered when developing follow-up protocols for survivors of childhood cancer.

## Data Availability

The datasets used and analysed during the current study are available from the corresponding author on reasonable request.

## Abbreviations

ALL: Acute Lymphoblastic Leukaemia
BT: Brain Tumour
PedsQL™: Pediatric Quality of Life
